# Supplementary material for: Reconciling Mining with the Conservation of Cave Biodiversity: A Quantitative Baseline to Help Establish Conservation Priorities
Source: PLoS One. 2016 Dec 20;11(12):e0168348. doi: 10.1371/journal.pone.0168348 (PMC5173368; doi:10.1371/journal.pone.0168348)
Supplement: S1 Dataset — (ZIP) [file pone.0168348.s002.zip › Taxa/Serra Norte/SN_2013/N5S/Lista N5S-70.pdf]

CAVIDADE N5S-0070

Classe	Ordem	Fam/Outros	Gên/Outros	Espécie	S-U	U-S	Zon.
Arachnida	Acari	Asternoseiidae		sp.84	X		P
Arachnida	Acari	Erythraeidae		sp.1		X	P
Arachnida	Acari	Laelapidae	*Stratiolaelaps*	sp.1		X	A
Arachnida	Acari	Mesostigmata		jovem	X		P
Arachnida	Acari	Neothyridae	*Diplothyrus*	sp.1		X	A
Arachnida	Acari	Sphaerolichidae		sp.1		X	A
Arachnida	Acari	Trombiculidae		sp.39		X	A
Arachnida	Acari	Trombidiformes		sp.49	X		P
Arachnida	Amblypygi	Charinidae	*Charinus*	sp.1	1	1	P
Arachnida	Amblypygi	Phrynidae	*Heterophrynus*	*longicornis*	5	4	P/A
Arachnida	Araneae	Caponiidae		sp.1	X	X	P/A
Arachnida	Araneae	Ctenidae		jovem		1	
Arachnida	Araneae	Ochyroceratidae	*Ochyrocera*	sp.3	X	X	P/A
Arachnida	Araneae	Pholcidae	*Leptopholcus*	sp.1		X	E
Arachnida	Araneae	Pholcidae	*Mesabolivar*	sp.1		X	E
Arachnida	Araneae	Prodidomidae		jovem		X	P
Arachnida	Araneae	Scytodidae	*Scytodes*	jovem		1	E
Arachnida	Araneae	Theraphosidae		jovem	1		P
Arachnida	Araneae	Theridiidae	*Theridion*	sp.1		X	E
Arachnida	Araneae	Theridiosomatidae	*Plato*	sp.1	X	X	P/A
Arachnida	Opiliones	Escadabiidae		sp.1	X		P
Arachnida	Palpigradi	Eukoeneniidae	*Allokoenenia*	sp.1		X	P
Arachnida	Pseudoscorpiones	Chernetidae	*Spelaeochernes*	sp.1	X		P/A
Arachnida	Pseudoscorpiones	Chthoniidae		jovem	X	X	P
Chilopoda	Scutigeromorpha	Pselliodidae	*Sphendononema*	jovem	1	2	P
Diplopoda	Glomeridesmida	Glomeridesmidae		sp.1		1	A
Diplopoda	Polydesmida	Chelodesmidae		jovem	1		P
Diplopoda	Polydesmida	Pyrgodesmidae		sp.1	X	X	A
Diplopoda	Spirostreptida	Pseudonannolenidae	*Pseudonannolene*	sp.2	1		P
Entognatha	Collembola	Sminthuroidea		sp.2	X	X	P/A
Entognatha	Diplura	Campodeidae		sp.1	X	X	E
Entognatha	Diplura	Japygidae		sp.3		X	P
Insecta	Blattodea	Blaberidae		sp.1	4		P
Insecta	Blattodea	Blattellidae		sp.5	4		P
Insecta	Blattodea	Blattidae		sp.5	3		P
Insecta	Blattodea	Polyphagidae		sp.1		3	P
Insecta	Coleoptera	Carabidae	*Coarazuphium*	sp.n.1	X		A
Insecta	Coleoptera	Scydmaenidae		sp.9	X		P
Insecta	Coleoptera	Staphylinidae	Pselaphinae	sp.3		X	A
Insecta	Diptera	Cecidomyiidae	Cecidomyiinae	sp.	X	X	E/P
Insecta	Diptera	Chironomidae		sp.	X	X	P/A
Insecta	Diptera	Dolichopodidae		sp.	X		P
Insecta	Diptera	Psychodidae	Phlebotominae	sp.	X	X	E/P/A
Insecta	Diptera	Sciaridae		sp.	X		P
Insecta	Diptera	Tipulidae	Tipulinae	sp.		X	E
Insecta	Heteroptera	Reduviidae	Emesinae	sp.		2	E/A
Insecta	Heteroptera	Reduviidae	*Panstrongylus*	sp.1	5		P
Insecta	Heteroptera	Reduviidae	Reduviinae	sp.	2		E
Insecta	Heteroptera	Reduviidae	Triatominae	sp.	3	1	A

Insecta	Homoptera	Cixiidae		sp.5	X	X	P
Insecta	Hymenoptera	Formicidae	*Gnamptogenys*	sp.2	X		P
Insecta	Hymenoptera	Formicidae	*Hypoponera*	sp.1		X	A
Insecta	Hymenoptera	Formicidae	*Paratrechina*	sp.1	X		P
Insecta	Hymenoptera	Formicidae	*Paratrechina*	sp.2	X		P
Insecta	Hymenoptera	Vespidae		sp.1		X	E
Insecta	Isoptera	Termitidae	*Convexitermes*	sp.	X	X	E/P
Insecta	Isoptera	Termitidae	*Nasutitermes*	sp.	X		P
Insecta	Lepidoptera	Gelechioidea		sp.		X	E/P
Insecta	Orthoptera	Phalangopsidae	*Paraclodes*	sp.	4		P
Insecta	Orthoptera	Phalangopsidae	*Phalangopsis*	sp.	23	38	E/P/A
Insecta	Thysanura	Ateluridae		sp.1	X	X	P
Insecta	Thysanura	Nicoletiidae		sp.1	X	X	P
Gastropoda	Pulmonata	Systrophiidae	*Happia*	sp.		X	P
Symphyla		Scutigerellidae		sp.		X	P
Amphibia	Anura	Strabomantidae	*Pristimantis*	cf. *fenestratus*	6	2	E/P
Mammalia	Rodentia	Cricetidae	*Rhipidomys*	sp.	1		A
